# Ruthenium(III) Complexes of Heterocyclic Tridentate (ONN) Schiff Base: Synthesis, Characterization and its Biological Properties as an Antiradical and Antiproliferative Agent

**DOI:** 10.3390/ijms17010060

**Published:** 2016-01-04

**Authors:** Ikechukwu P. Ejidike, Peter A. Ajibade

**Affiliations:** Department of Chemistry, Faculty of Science and Agriculture, University of Fort Hare, P.B. X1314, Alice 5700, South Africa; pejidike@ufh.ac.za

**Keywords:** tridentate Schiff base, heterocyclic Ru(III) complexes, spectroscopy, antiradical, antiproliferative

## Abstract

The current work reports the synthesis, spectroscopic studies, antiradical and antiproliferative properties of four ruthenium(III) complexes of heterocyclic tridentate Schiff base bearing a simple 2′,4′-dihydroxyacetophenone functionality and ethylenediamine as the bridging ligand with RCHO moiety. The reaction of the tridentate ligands with RuCl_3_·3H_2_O lead to the formation of neutral complexes of the type [Ru(L)Cl_2_(H_2_O)] (where L = tridentate NNO ligands). The compounds were characterized by elemental analysis, UV-vis, conductivity measurements, FTIR spectroscopy and confirmed the proposed octahedral geometry around the Ru ion. The Ru(III) compounds showed antiradical potentials against 2,2-Diphenyl-1-Picrylhydrazyl (DPPH) and 2,2′-azinobis-(3-ethylbenzothiazoline-6-sulfonic acid) (ABTS) radicals, with DPPH scavenging capability in the order: [(PAEBOD)RuCl_2_] > [(BZEBOD)RuCl_2_] > [(MOABOD)RuCl_2_] > [Vit. C] > [rutin] > [(METBOD)RuCl_2_], and ABTS radical in the order: [(PAEBOD)RuCl_2_] < [(MOABOD)RuCl_2_] < [(BZEBOD)RuCl_2_] < [(METBOD)RuCl_2_]. Furthermore, *in vitro* anti-proliferative activity was investigated against three human cancer cell lines: renal cancer cell (TK-10), melanoma cancer cell (UACC-62) and breast cancer cell (MCF-7) by SRB assay.

## 1. Introduction

There have been different reports on the preparation and spectral analysis of various forms of Schiff base ligands, including bi-, tri-, tetra- and polydentate incorporating transition and non-transition metals [1,2,3]. Schiff base-transition metal complexes obtained from heterocyclic molecules have received attention from many researchers regarding the development of bioinorganic compounds for biological application [4]. Biologically active metal-complexes bearing Schiff base derived from vinyl aniline and heterocyclic aldehydes with octahedral geometry have been reported to exhibit promising antimicrobial activities due to the chelation process dominantly affecting the general biological performance of the synthesized compounds [5]. Metal complexes-DNA interaction studies have triggered researchers′ attention owing to their applications in the planning and chemotherapeutic agents improvement, synthetic control of enzymes [6], DNA-cleavage agents and DNA ”molecular light switches” [7,8,9] because of their potential to bind DNA and cleave the duplex [10,11]. Radical species have been associated with several oxidative damages diseases such as liver cirrhosis, atherosclerosis, cancer, diabetes, and ageing [12]. The steady increase in free radical production usually leads to cell wall and DNA damage, translating to chronic diseases such as cancers and other related disease, especially in disproportionate concentrations in human cells as the system might not be able to safeguard against its consequences [12,13].

Antioxidants derived from metal coordination have gained attention recently concerning safeguarding living organisms and cells from damage associated with oxidative stress or free radicals [14]. The biological efficiencies of transition metal complexes have been investigated through different techniques such as the hydroxyl radical scavenger, the superoxide anion radical scavenger, and superoxide dismutase [15]. The Schiff base of ruthenium complexes is one of the compounds that have attracted great attention, and some of its complexes have exhibited interesting properties such as intracellular accumulation and antiproliferative properties [16,17,18] and also their unique luminescence properties when bound to DNA [19]. Ru(III) complexes with low spin stability of the type [RuX_2_(EPh_3_)_2_(L)] (where E = P or As; X = Cl or Br; L = mono basic bidentate Schiff bases) inhibited the growth of *Staphylococcus aureus* (209p) and *E. coli ESS* (2231) [20]. Tetradentate Schiff base of Ru(III) complexes incorporating N_2_O_2_ donors have been reported to exhibit moderate to strong scavenging activity on DPPH and ABTS radicals and low to moderate antiproliferative effect against some selected cancer cell lines [21]. In continuation of our efforts towards the synthesis of coordination compounds with potential chemotherapeutic properties [22], we report the synthesis, characterization and biological studies of Ru(III) tridentate Schiff base ligand formulated as [Ru(L)Cl_2_(H_2_O)] (where L = tridentate ONN Schiff base ligand). The compounds were characterized by elemental analysis, electronic and infrared spectroscopic techniques. The biological properties were evaluated to determine their radical scavenging potentials and *in vitro* anticancer studies against three human cancer cell lines.

## 2. Results and Discussion

### 2.1. Synthesis

**Scheme 1:**
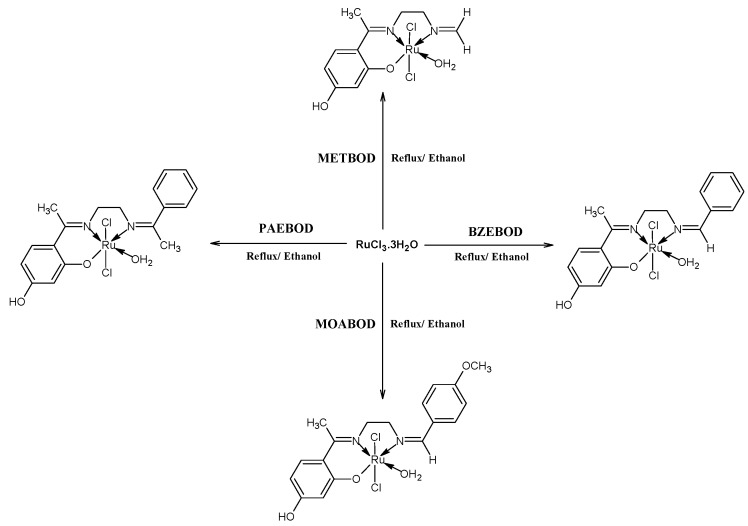
Synthetic pathway for the heterocyclic Ru(III) complexes.

In line with the study, 15 mmol in 20 mL ethanol was added dropwise to 30 mL ethanol solution containing 2′,4′-Dihydroxyacetophenone, substituted aldehydes and ethylenediamine, acting as the bridging ligand in ethanol afforded the desired heterocyclic ONN Schiff base ligands (METBOD, BZEBOD, MOABOD, PAEBOD), which was reacted with RuCl_3_·3H_2_O to give the corresponding heterocyclic Ru(III) compounds. Analytical and spectroscopic data were in good conformity with the proposed structure of the Ru(III) complexes as shown in Scheme 1. [Ru(L)Cl_2_(H_2_O)] (where L = tridentate heterocyclic Schiff base ligand). The isolated Ru(III) compounds were non-electrolyte in solution with molar conductance (*Λµ*) in 10^−3^ mol/L DMF solution in the range 23.8–47.4 µScm^−1^ [23].

### 2.2. Infrared Spectral Studies of the Ru(III) Complexes

The infrared spectra of the free ligand and the heterocyclic Ru(III) compounds were compared and carefully assigned. The Schiff base ligand showed broad bands in the 3473–3470 cm^−1^ region, which is attributable to the ν(OH) cm^−1^ stretching vibrations. In the heterocyclic Ru(III) compounds, these stretching vibrations due to the OH modes were not observed, suggesting the displacement of the hydroxyl proton by Ru^3+^ ion leading to covalent ν(Ru–O) bonding with the ligand [24]. This was further supported by the strong band observed in the free Schiff base in the range 1254–1168 cm^−1^ and due to phenolic ν(C–O) stretching vibrations. In all the Ru(III) complexes, these bands shifted to higher wavenumbers in the range 1284–1172 cm^−1^, confirming the coordination of the ruthenium ion through the phenolic oxygen atom [25,26]. Stretching vibrations due to the coordinated water in the heterocyclic Ru(III) complexes were observed in the regions 3449–3422 and 854–810 cm^−1^. These vibrations are assigned to symmetric and antisymmetric ν(O–H) stretching and ν(O–H) rocking vibrations, which further confirmed the coordination of non-ligand due to the rocking mode of water [22,27] while those above 3500 cm^−1^ are due to free OH [28]. The ν(CH=N) of the heterocyclic Ru(III) compounds showed a strong band in the region 1629–1620 cm^−1^ [14,29]. The shifting of this band to higher vibration frequency by 12–14 cm^−1^ confirms the coordination of the nitrogen atom of the azomethine group to the Ru(III) ion [21]. The bonding of the Ru^3+^ ions to the METBOD, BZEBOD, MOABOD, PAEBOD through the (>C=N) nitrogen and phenolic oxygen atoms is further confirmed through the appearance of new bands in the 520–477 and 476–418 cm^−1^ range due to the ν(Ru–N) and ν(Ru–O) vibrations, respectively [21,24].

### 2.3. Electronic Absorption Spectra Studies of Heterocyclic Ru(III) Compounds

The electronic absorption spectra of heterocyclic Ru(III) complexes in DMF within the range of 900–200 nm showed four to five bands within the region 15,748–36,101 cm^−1^. Ruthenium(III) ground state is ^2^T_2g_ and the first excited doublet levels in the order of increasing energy are ^2^A_2g_ and ^2^T_1g_, which arise from $t_{2g}^{4}e_{g}^{1}$ configuration [30]. Ligand-centered transitions ranged from 24,938–36,101 cm^−1^ in the spectral sketches, and the these bands are attributable to π* ← π and π* ← n transitions of the aryl ring and the double bond of the >C=N– group [20,21]. The ruthenium(III) ion, with a d^5^ electronic configuration, has relatively high oxidizing properties and a large crystal field parameter, and the band charge transfer of the type L_πy_ → T_2g_ is noticeable in the low energy region, which obscures the weaker bands due to *d-d* transitions [20,21]. The band in the 15,748–19,763 cm^−1^ region have been assigned to the ^2^T_2g_ → ^2^A_2g_ transition, which is in conformity with the assignments made for similar ruthenium(III) complexes [31]. Absorption in the 19,343–25,576 cm^−1^ region displayed bands assignable to the charge transfer transitions [32]. The design of the absorption spectra for the heterocyclic Ru(III) complexes confirm the proposed octahedral environment around the ruthenium(III) ion [21].

### 2.4. The Antioxidant Assay

Reactive oxygen species (ROS) have been reported to be a significant promoter of cellular damage of biomolecules, organelles and invariably lead to several diseases such as Parkinson’s disease, aging, heart disease, and cancer [12,13,14]. Various sample concentrations in DMF as solvent were used to carry out the antioxidant study, whereas standards include Vitamin C, butylated hydroxytoluene (BHT), and rutin hydrate (Rutin). As such, the capacity of the heterocyclic Ru(III) complexes to scavenge radicals have been investigated using the DPPH* and ABTS*^+^ radicals (Table 1).

**Table 1:** The IC_50_ values of DPPH- and ABTS-scavenging activity of heterocyclic Ru(III) complexes compared to a standard anti-oxidant drug.

Compounds	DPPH Radical Activity		ABTS Radical Activity	
	IC_50_ (µM)	*R*^2^	IC_50_ (µM)	*R*^2^
[(METBOD)RuCl_2_] (**1**)	2.86 ± 0.57	0.971	2.98 ± 1.44	0.878
[(BZEBOD)RuCl_2_] (**2**)	1.52 ± 0.36	0.936	3.28 ± 1.26	0.967
[(MOABOD)RuCl_2_] (**3**)	1.55 ± 0.54	0.973	3.29 ± 0.94	0.917
[(PAEBOD)RuCl_2_] (**4**)	1.50 ± 0.40	0.960	3.54 ± 1.31	0.812
Vitamin C *	1.92 ± 1.07	0.978	-	-
Rutin *	2.52 ± 1.60	0.798	2.83 ± 1.84	0.983
BHT *	-	-	1.64 ± 1.54	0.919

(*n* = 3, X ± SEM), IC_50_: Inhibitory concentration; shows the percent inhibition of the examined compound at 50%, *R*^2^: correlation coefficient; (*) Standards; (-) No result.

#### 2.4.1. (DPPH) Free Radical Scavenging Activity (FRSA) Assay

**Figure 1:**
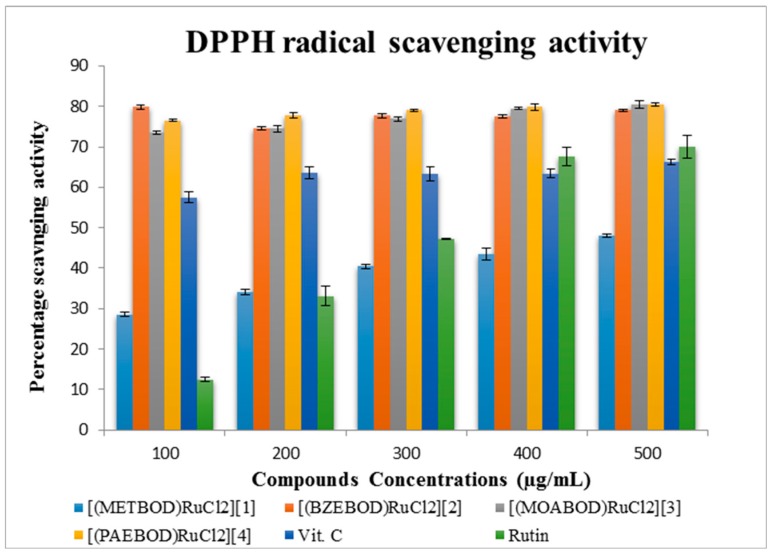
Heterocyclic Ru(III) complex and standard drug DPPH scavenging action.

The DPPH scavenging effect is based on the absorbance decrease of alcoholic DPPH solution in the presence of proton releasing species [33]. Activities of heterocyclic Ru(III) complexes solution; ascorbic acid (Vit. C) and rutin as standards are shown in Figure 1. The ligand *viz*. METBOD, BZEBOD, MOABOD, PAEBOD shows trivial DPPH activity; however, upon coordination with Ru^3+^ ion, the scavenging properties were significantly improved, thereby making the Ru(III)-tridentate Schiff base complexes more effective DPPH radical scavengers than the analogous free Schiff base. The observed DPPH activities of the tested samples possess strong electron donating power as compared to those of the standards (ascorbic acid and rutin). IC_50_ and its corresponding *R*^2^ (correlation coefficient) values of tested compounds are listed in Table 1. Ru(III)-tridentate Schiff base complexes, alongside with Vit. C and rutin DPPH scavenging capability can be ranked in the following order: [(PAEBOD)RuCl_2_] > [(BZEBOD)RuCl_2_] > [(MOABOD)RuCl_2_] > [Vit. C] > [rutin] > [(METBOD)RuCl_2_]. Compounds [(PAEBOD)RuCl_2_], [(BZEBOD)RuCl_2_] and [(MOABOD)RuCl_2_] with IC_50_ values of 1.50 ± 0.40, 1.52 ± 0.36 and 1.55 ± 0.54 µM, respectively, exhibited higher scavenging activity against DPPH than the commercially available Vit. C and rutin (standard); however, [(METBOD)RuCl_2_] showed the lowest activity of all investigated samples with an IC_50_ value of 2.86 ± 0.57 µM. In addition, the isolated Ru(III)-tridentate Schiff base complexes were found effective as DPPH scavengers at different concentrations, thereby making them potential compounds for developing anti-stress inducing agents [22].

#### 2.4.2. ABTS Scavenging Property of Heterocyclic Ru(III) Compounds

**Figure 2:**
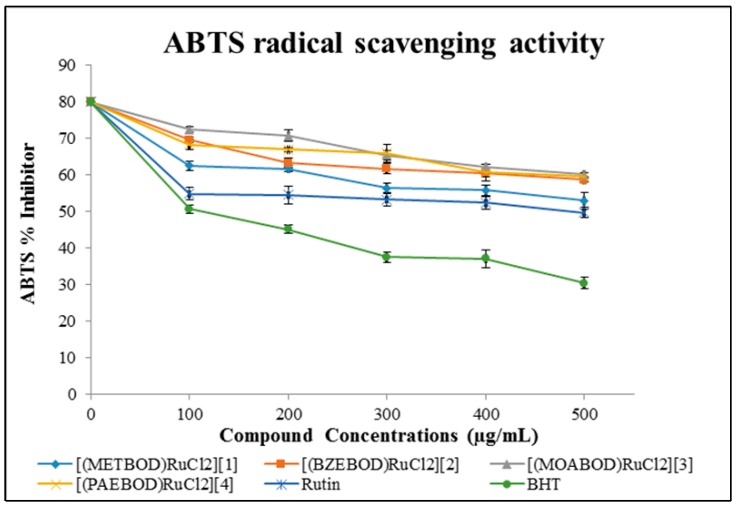
ABTS activities of heterocyclic Ru(III) complexes and standard drugs.

The antioxidant potentials of the heterocyclic Ru(III) complexes in this study was further confirmed by examining their ABTS capability. The outcome of the Ru(III) complexes on 2,2′-azinobis-(3-ethylbenzothiazoline-6-sulfonic acid) (ABTS^+^) radicals are presented in Table 2. At 734 nm, the absorbance of active ABTS*^+^ solution [34] obviously declined upon the addition of different concentrations of heterocyclic Ru(III) complexes, and the same trend was also observed for the standard drugs with the percentage ABTS inhibition as presented in Figure 2.

**Table 2:** IC_50_ values (µM) of heterocyclic Ru(III) compounds and Parthenolide against human cell lines.

Anticancer Activity IC_50_ (µM) 48 h				
Compounds	Molecular Formula	TK-10	UACC-62	MCF-7
[(BZEBOD)RuCl_2_] (**2**)	C_17_H_21_N_2_O_4_RuCl_2_	10.34 ± 1.35	6.63 ± 1.92	3.63 ± 1.92
[(MOABOD)RuCl_2_] (**3**)	C_18_H_23_N_2_O_5_RuCl_2_	14.47 ± 0.98	6.27 ± 0.89	3.99 ± 1.45
[(PAEBOD)RuCl_2_] (**4**)	C_18_H_23_N_2_O_4_RuCl_2_	11.85 ± 4.50	4.88 ± 0.53	3.79 ± 3.03
Parthenolide * (D)	C_15_H_20_O_3_	0.50 ± 1.43	0.89 ± 2.18	0.44 ± 2.02

(*) Standard; Cells were treated with various concentrations of compounds required to inhibit 50% of the culture growth when exposed for 48 h (IC_50_ values was obtained). Each value represents the mean ± SD of three independent experiments.

The effectiveness of the tested samples with highest inhibition in quenching ABTS*^+^ in the system was identified at the lowest concentration (100 µg/mL) with the metal complexes exhibiting higher % inhibition than the standards. However, compound [(METBOD)RuCl_2_] showed significantly higher ABTS scavenging activity with an IC_50_ value of 2.98 ± 1.44 µM while complexes of [(BZEBOD)RuCl_2_], [(MOABOD)RuCl_2_] and [(PAEBOD)RuCl_2_] gave an IC_50_ value of 3.28 ± 1.26, 3.29 ± 0.94, 3.54 ± 1.31 µM respectively. The scavenging activity pattern of the complexes on ABTS radicals is in the following order: [(PAEBOD)RuCl_2_] < [(MOABOD)RuCl_2_] < [(BZEBOD)RuCl_2_] < [(METBOD)RuCl_2_]. It can be concluded that the heterocyclic Ru(III) complexes showed better DPPH scavenging properties than that of ABTS radicals, hence making the compounds worthwhile therapeutic agents for developing compounds for averting oxidative cell damage, as various free radicals are generated in the system promoting cancer, aging and cardiovascular diseases [21,34].

### 2.5. Anti-Proliferative Activity Evaluation

**Figure 3:**
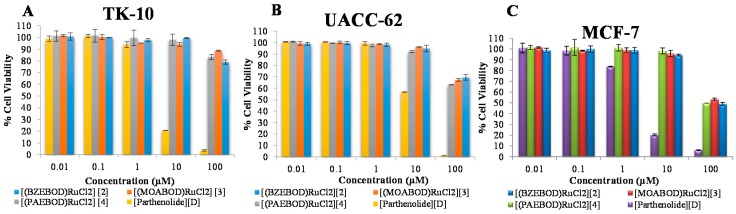
Antiproliferative measurements of heterocyclic ruthenium(III) complexes graphs [2,3,4], parthenolide [D] in **A**–**C** representing cell viability charts for the corresponding Ru(III) complexes against the selected cancer cell lines. (**A**) Human renal cancer cell (TK-10); (**B**) Human melanoma cancer cell (UACC-62); (**C**) Human breast cancer cell (MCF-7).

The biochemical actions of Ru(III)-tridentate Schiff base complexes were analyzed, in order to investigate the structure-activity relationship of the isolated compounds with respect to different characteristic reactive atoms (functional groups). Three of the heterocyclic Ru(III) compounds were subjected to cytotoxicity tests using different sample concentrations towards human renal cancer cell (TK-10), human melanoma cancer cell (UACC-62) and human breast cancer cell (MCF-7). The tumor cell lines were incubated for 48 h, followed by the addition of the compounds at various concentrations, and then subjected to the Sulforhodamine B (SRB) assay [21]. Parthenolide served as positive control. The percentage of cell viability was plotted as a function of heterocyclic ruthenium(III) complex concentration as presented in Figure 3A–C. IC_50_ values are summarized in Table 2. The results obtained from this study demonstrated that treatment of cells with different heterocyclic Ru(III) complexes, efficiently affected cell viability toward MCF7 cells, as displayed in Figure 3 and Table 2. Parthenolide exhibited strong levels of antiproliferative activity against the studied cell lines, in accordance with previous reports [35,36]. The Ru(III) compounds exhibited low to moderate *in vitro* antiproliferative activities against the selected cell lines as compared to the standard drug (Parthenolide). [(BZEBOD)RuCl_2_], [(MOABOD)RuCl_2_] and [(PAEBOD)RuCl_2_] induced more efficient cell death with IC_50_ values of 3.63 ± 1.92, 3.99 ± 1.45, and 3.79 ± 3.03 μM, respectively, towards MCF7 cells than other investigated cell lines (Figure 3C and Table 2).

In contrast, the compound induced a weak effect on human renal cancer cell (TK-10), while compound [(PAEBOD)RuCl_2_] induced efficient cell death on human melanoma cancer cells (UACC-62) with an IC_50_ value of 4.88 ± 0.53 μM; [(BZEBOD)RuCl_2_] and [(MOABOD)RuCl_2_] showed moderate inhibition with IC_50_ values of 6.63 ± 1.92 and 6.27 ± 0.89 μM. In all, the orders of cytotoxicity of the compounds are in the following order: [(BZEBOD)RuCl_2_] > [(PAEBOD)RuCl_2_] > [(MOABOD)RuCl_2_]. This activity could be based on the nature of substituents, hydroxyl, alkyl and methoxy groups and ethylenediamine, acting as bridging spacers playing significant roles in antiproliferative of Ru(III)-tridentate Schiff base complexes. The results of *in vitro* evaluation gave some insight into the structure-activity relationship, but the overall anti-proliferative activity of the metal complexes usually depends on various factors, including complex/compound reactivity, intrinsic structure, cellular uptake potential, and interaction of the cells [37].

## 3. Experimental Section

### 3.1. Materials and Methods

Analytical grade solvents and chemicals were used as obtained without further purification. Ascorbic acid and dimethyl sulfoxide (DMSO) were obtained from Merck (Johannesburg, South Africa), butylated hydroxytoluene, 2,2′-azinobis-3-ethylbenzothiazoline-6-sulfonic acid (ABTS), Rutin hydrate, 2,2-diphenyl-1-picrylhydrazyl (DPPH), were received from Sigma Chemical Co., (St. Louis, MO, USA). Elemental analysis data were obtained on Perkin Elmer elemental analyzer (Massachusetts, UK). Newly prepared 10^−3^ M DMF with Crison EC-Meter Basic 30+ conductivity cell (Alella-Barcelona, Spain) were used for the conductivity measurements. Perkin Elmer-FT-IR spectrometer (Spectrum 2000, London, UK) within the range 4000–400 cm^−1^ using KBr pellets was used for IR spectra data collection. Absorption data were documented with Perkin Elmer Lambda-25 UV-visible spectrometer (Waltham, MA, USA), ranging from 200–900 nm in quartz cells of path length 1 cm. The Stuart melting point (SMP 11, Staffordshire, UK) was used for the melting points determination. RuCl_3_·3H_2_O was obtained from Aldrich (Johannesburg, South Africa). Four tridentate ligands, *viz*. 4-{(1*E*)-*N*-[2-(methylideneamino)ethyl]ethanimidoyl}benzene-1,3-diol [METBOD], 4-[(1*E*)-*N*-{2-[(Z)-benzylideneamino]ethyl}ethanimidoyl]benzene-1,3-diol [BZEBOD], 4-[(1*E*)-*N*-{2-[(Z)-(4-methoxybenzylidene)amino]ethyl}ethanimidoyl]benzene-1,3-diol [MOABOD], 4-[(1*E*)-*N*-{2-[(Z)-(1-phenylethylidene)amino]ethyl}ethanimidoyl]benzene-1,3-diol [PAEBOD] were synthesized and reported. 

### 3.2. Synthetic Procedure for Heterocycles (METBOD, BZEBOD, MOABOD, PAEBOD)

In a 250 mL round bottom flask, ethylenediamine (15 mmol) in 20 mL ethanol was slowly added to ethanol solution (30 mL) containing 2′,4′-Dihydroxyacetophenone (15 mmol), allowed to stir for 1 h at room temperature, then followed by drop-wise addition for 10–15 min of applicable aldehyde (15 mmol) dissolved in 20 mL ethanol at room temperature. The subsequent mixture was refluxed for 3–4 h, and further stirred for 22–24 h at room temperature to give the desired tridentate compounds as crystalline solid. The crude product was recrystallized from warm ethanol and dried in the vacuum at 50 °C overnight to give analytically pure products in good to excellent yields (65.7% to 88.8%).

### 3.3. General Procedure for the Synthesis of Ru(III) Compounds

RuCl_3_·3H_2_O (0.5 mmol, 103.7 mg) in 15 mL of absolute ethanol, was added into a warm ethanolic solution (20 mL) of (0.5 mmol); 103.1 mg of METBOD, 141.2 mg of BZEBOD, 156.2 mg of MOABOD, 148.2 mg of PAEBOD in equal molar ratio. The colour of the solutions changed immediately, and they were magnetically stirred and refluxed for 6 h. The obtained solids were filtered by suction from the reaction medium, purified with ethanol followed by diethyl ether, and dried over dry CaCl_2_.

**[(METBOD)RuCl_2_(H_2_O)]·2H_2_O (1):** Brownish-green Solid; Yield: 127.3 mg (59.04%); Decomp. Temp, °C, 211–212 °C; Conductivity (µScm^−1^): 41.2; IR (KBr) ν_max_/cm^−1^: 3422 (O–H), 1622 (C=N), 1284, 1173 (C–O), 477 (Ru–N), 418 (Ru–O); UV-Vis (DMF): λ_max/_nm (cm^−1^): 311 (32,155), 343 (29,155), 381 (26,247), 391 (25,576), 506 (19,763); Anal. Calcd. for C_11_H_19_N_2_O_5_RuCl_2_ (%): C: 30.64, H: 4.44, N: 6.50; Found (%): C: 30.41, H: 4.28, N: 6.23.

**[(BZEBOD)RuCl_2_(H_2_O)]·1H_2_O (2):** Darkish-green Solid; Yield: 161.5 mg (66.0%); Decomp. Temp, °C, 228–229 °C; Conductivity (µScm^−1^): 32.0; IR (KBr) ν_max_/cm^−1^: 3442 (O–H), 1629 (C=N), 1249, 1172 (C–O), 520 (Ru–N), 435 (Ru–O); UV-Vis (DMF): λ_max/_nm (cm^−1^): 280 (35,715), 308 (32,468), 389 (25,707), 514 (19,343), 623 (16,051); Anal. Calcd. for C_17_H_21_N_2_O_4_RuCl_2_ (%): C: 41.73, H: 4.33, N: 5.72; Found (%): C: 42.01, H: 4.18, N: 5.51.

**[(MOABOD)RuCl_2_(H_2_O)]·1H_2_O (3):** Darkish-green Solid; Yield: 177.3 mg (68.3%); Decomp. Temp, °C, 232–233 °C; Conductivity (µScm^−1^): 45.2; IR (KBr) ν_max_/cm^−1^: 3449 (O–H), 1621 (C=N), 1250, 1181 (C–O), 519 (Ru–N), 476 (Ru–O); UV-Vis (DMF): λ_max/_nm (cm^−1^): 282 (35,461), 326 (30,675), 384 (26,042), 401 (24,938), 517 (19,343), 635 (15,748); Anal. Calcd. for C_18_H_23_N_2_O_5_RuCl_2_ (%): C: 41.63, H: 4.46, N: 5.39; Found (%): C: 41.51, H: 4.70, N: 5.25.

**[(PAEBOD)RuCl_2_(H_2_O)]·1H_2_O (4):** Darkish-green Solid; Yield: 159.6 mg (63.4%); Decomp. Temp, °C, 231–232 °C; Conductivity (µScm^−1^): 34.7; IR (KBr) ν_max_/cm^−1^: 3418 (O–H), 1620 (C=N), 1251, 1173 (C–O), 481 (Ru–N), 427 (Ru–O); UV-Vis (DMF): λ_max/_nm (cm^−1^): 277 (36,101), 312 (32,052), 378 (26,455), 394 (25,381), 511 (19,570), 631 (15,848); Anal. Calcd. for C_18_H_23_N_2_O_4_RuCl_2_ (%): C: 42.95, H: 4.61, N: 5.57; Found (%): C: 43.14, H: 4.82, N: 5.35.

### 3.4. The Antioxidant Assay

#### 3.4.1. 2,2-Diphenyl-1-picrylhydrazyl (DPPH) Free Radical Scavenging Activity (FRSA) Assay

The antioxidant activity of the synthesized compounds were determined using a stable 2,2-Diphenyl-1-picrylhydrazyl (DPPH) reagent following a method that has been reported previously [14]. DMF solutions (1 mL) of the samples with varying concentrations (100, 200, 300, 400 and 500 µg/ mL) was vortex thoroughly with 1 mL of methanolic solution of 0.4 mM DPPH and allowed to interact for about 30 min in the dark. Reduction in absorption of the solutions was measured spectrophotometrically at 517 nm against the control. The equation below has been used to obtain the percentage of scavenged DPPH radical:

(1) $$\text{Percentage scavenging activity}=\frac{\text{ Absorbance of control}-\text{ Absorbance of sample}}{\text{Absorbance of control}}\;\times \;100$$

#### 3.4.2. ABTS Radical Scavenging Prospects

The ruthenium(III) compound’s 2,2′-azinobis-(3-ethylbenzothiazoline-6-sulfonic acid) (ABTS) scavenging ability adopted a previously described method [22]. Two stock solutions in equal amounts, 7 mM ABTS solution and 2.4 mM K_2_S_2_O_8_ solution, were mixed to obtain the working solution and left in the dark for 12 h for complete reaction. In order to achieve spectrophotometric absorbance of 0.706 ± 0.001 units at 734 nm, 1 mL of ABTS^+^ was diluted. The scavenging properties of the samples were determined [22] with standard drugs as butylated hydroxyl toluene and rutin hydrate. Triplicates analysis was carried out and we averaged the results. The ABTS percentage inhibition calculated was determined by following the equation:

(2) $$(\%)\text{ABTS Inhibition}=\frac{\text{ Absorbance of control}-\text{ Absorbance of sample}}{\text{Absorbance of control}}\;\times \;100$$

### 3.5. Cell Viability Assay

The SRB assay was used for *in vitro* anticancer study of the synthesized compounds was done as previously described [21]. The human renal cancer cell line (TK-10), human melanoma cancer cell line (UACC-62) and human breast cancer cell line (MCF-7) were cultured at 37 °C with 95% air, 5% CO_2_ and 100% relative humidity in RPMI medium, and supplemented with 5% fetal bovine serum (FBS), 50 μg·mL^−1^ (gentamicin) and 2 mM l-glutamine as described [38]. 3–19 passages were inoculated into 96-well microtitre plates at plating densities of 7–10,000 cells/well and were incubated for 24 h. Treatment of the cells with the solutions of compounds in DMSO was done after 24 h, and watered down in medium to yield 5 different concentrations of 0.01, 0.1, 0, 10 and 100 μM, while cells that contained no drug/sample were used as control and blanks comprised complete medium with no cells. The standard used for this study was the parthenolide. Incubation of plates for 48 h was followed with addition of the compounds. Viable cells were fixed to the bottom of each well with cold 50% trichloroacetic acid, and washed, dried and dyed by SRB. The boundless dye was detached, while 10 mM Tris base was used for the extraction of protein-bound dye, and its optical density determination achieved using a multi-well spectrophotometer at the wavelength 540 nm. 50% of cell growth inhibition was calculated by non-linear regression, as absorbance values were plotted against concentration of compounds to determine the IC_50_. In order to ensure the quality of immunocytochemical assays such as the Sulforhodamine B (SRB), the Z′-factor coefficient was adapted.

## 4. Conclusions

Four heterocyclic ruthenium(III)-tridentate Schiff base complexes formulated as [Ru(L)Cl_2_(H_2_O)] (where L = tridentate ONN Schiff base ligand) were synthesized and characterized using spectroscopic and analytical techniques. The microanalyses were in good agreement with the proposed structures of the compounds. The absorption spectra revealed that the geometry around the Ru^3+^ ion in the monomeric complex is octahedral, in which the ligands act as tridentate chelating ligands, coordinating through azomethine nitrogen atoms and a phenol oxygen atom. All the ruthenium(III) complexes were effective as radical scavengers at different concentrations, thereby making them potential compounds for developing anti-stress inducing agents with DPPH scavenging capability in the following order: [(PAEBOD)RuCl_2_] > [(BZEBOD)RuCl_2_] > [(MOABOD)RuCl_2_] > [Vit. C] > [rutin] > [(METBOD)RuCl_2_] and ABTS radical in the order: [(PAEBOD)RuCl_2_] < [(MOABOD)RuCl_2_] < [(BZEBOD)RuCl_2_] < [(METBOD)RuCl_2_]. Furthermore, the possible explanations on the structure-activity relationship for the mode of interaction of these complexes against the different tumor cell lines are exemplified and found to affect cell viability efficiently toward MCF-7 cells. The order of anti-proliferative activity with respect to Ru(III) complexes is as follows: [(BZEBOD)RuCl_2_] > [(PAEBOD)RuCl_2_] > [(MOABOD)RuCl_2_].
